# Humoral, cellular immunity and efficacy of bioreactor propagated and inactivated Fowl adenovirus 8b adjuvanted with Montanide 71VG in broiler chickens

**DOI:** 10.5455/javar.2024.k819

**Published:** 2024-09-29

**Authors:** Chidozie C. Ugwu, Mohd Hair-Bejo, Mat I. Nurulfiza, Abdul R. Omar, Aini Ideris

**Affiliations:** 1 Faculty of Veterinary Medicine, Universiti Putra Malaysia, Kuala Lumpur, Malaysia; 2 Department of Animal Science and Technology, Federal University of Technology, Owerri, Nigeria; 3 Institute of Bioscience, Universiti Putra Malaysia, Kuala Lumpur, Malaysia; 4 Faculty of Biotechnology and Biomolecular Sciences, University Putra Malaysia, Kuala Lumpur, Malaysia

**Keywords:** Fowl adenovirus, BEI, Montanide 71VG, Antibodies, T lymphocytes, Efficacy

## Abstract

**Objectives:**

The study aimed to inactivate the FAdV isolate (UPM11142P5B1) produced in a bioreactor and assess the humoral and cellular immunity, efficacy, and virus shedding in broiler chickens.

**Materials and Methods:**

The isolate was grown in a bioreactor, inactivated using binary ethyleneimine, adjuvanted with Montanide 71VG, and injected into day-old broiler chickens either with or without booster groups. The following parameters were measured: T lymphocyte profile in the liver, spleen, and thymus; FAdV antibody titer; clinical symptoms; gross and histological alterations in the liver, spleen, and thymus; virus copy number in the liver and cloacal shedding.

**Results:**

Compared to the unchallenged control group, booster (BG), and non-booster (NBG), the challenged control group (CCG) had a larger liver: body weight (BW) ratio, milder clinical signs, gross lesions, and histological alterations. They also had a lower BW. At 7, 21, 35, and 42 days post-inoculation (dpi), the NBG and BG exhibited higher antibody levels than the UCG. At 35 dpi, challenged BG and NBG produced more antibodies than CCG. In BG and NBG, T cells were stimulated in the spleen, thymus, and liver. At 35 and 42 dpi, the challenged BG and NBG showed significantly decreased viral copy numbers in the liver and shedding, respectively, along with increased lymphocyte counts.

**Conclusion:**

The inactivated UPM11142P5B1 with Montanide 71VG could be a vaccine against FAdV 8b infections in chickens.

## Introduction

Inclusion body hepatitis (IBH) is caused by the fowl adenovirus (FAdV) serotype 8b, which is a member of the genus Aviadenovirus and family Adenoviridae. This disease generates significant financial losses for the poultry industries globally [1,2]. Since the pathogen’s vertical spread makes control challenging, vaccination is the recommended course of action for its prevention. The age of the chicken and the method of inoculation may affect the strong humoral immune response elicited by inactivated FAdV vaccinations [3]. Nevertheless, although neutralizing antibodies might not be necessary to attain clinical protection, cellular immunity may be the major pathway to complete chicken protection against FAdV infection [4]. Research on cytokine expression patterns, which show a tilt towards the Th1-pathway upon infection with non-pathogenic FAdV-4 and -8b strains, has reinforced the significance of cell-mediated immunity (CMI) in limiting FAdV infections [5]. However, it is unclear how CMI contributes to FAdV 8b infection in chickens. Moreover, the inducement of CMI is typically not linked to inactivated vaccinations. However, complete protection by trivalent inactivated FAdV 4 and FAdV 8b/11 was reported [6], and vaccination of broiler chickens at 17 days old with inactivated FAdV 8b shielded them against pathogenic FAdV 8b challenge until 70 days old [7]. However, since the route to this protective efficacy was not entirely defined, CMI response could also play a role.

There are various ways to inactivate viruses, but the most used technique for producing vaccines is chemical treatment. An ideal inactivating agent would be binary ethyleneimine (BEI), an aziridine molecule that interacts with viral nucleic acids and preserves the epitope’s conformance and accessibility to a greater extent. BEI has been effectively utilized to inactivate viruses such as the rabies virus [8], Japanese encephalitis viruses [9], and foot and mouth disease viruses [10], but further research is needed to fully understand its applications. BEI inactivates viruses without affecting the antigenicity of the viral antigen.

Adjuvants are required after inactivation to promote immunogenicity and produce highly immunogenic inactivated vaccines. When given with adjuvants, inactivated vaccines produce the strongest antibody responses and the fullest protection against illness and virus shedding; similar results have been reported for Montanide 70VG and 71VG, which are useful adjuvants for chickens when tested with various bacterial and viral agents [11]. Its impact on chicken cellular immunity, however, is not well understood. The protective efficacy even with low humoral immunity may be explained by the lack of a thorough report on the induction of cellular immunity by inactivated FAdV 8b with Montanide 71VG in chickens, as far as we are aware. The purpose of this study was to evaluate the humoral, cellular immunity, effectiveness, and challenge viral shedding of inactivated FAdV 8b that was grown in a bioreactor and adjuvanted with Montanide 71VG in broiler chickens.

## Inoculum

The virus used was a FAdV 8b isolate UPM11142P5, which was five times serially passaged in chicken embryo liver (CEL) cells. Before use, the isolate was filtered using a 0.45 μm syringe filter (Corning, USA) and stored in the Makmal Virologi, Fakulti Perubatan Veterinar, Universiti Putra Malaysia.

### Challenge virus

FAdV serotype 8b isolate UPM11142P5, originally passaged 5x in CEL cells, was passaged 2x in embryonated chicken eggs to become UPM11142P5EP2. The supernatant of the embryonic liver homogenate that was passed through a 0.45 μm syringe filter and with an infective dose of EID50 of 10^8^/ml was used as a challenge virus [12].

### Bioreactor propagation

As previously reported [13], CEL cells were attached to Cytodex^TM^ 1 microcarrier and used to grow UPM11142P5 isolate 1x in a stirred tank bioreactor culture.

### Inactivation of FAdV virus and preparation of inoculum

1.5 l of viral supernatant was inactivated by adding 30 ml of 0.1 M BEI and mixing completely by vortexing. The mixture was incubated for 30 h at 37°C, and manually shaken every hour [14]. Following inactivation, 3 ml of 1M sodium thiosulfate was added, and the mixture was allowed to sit at room temperature for an hour. It was then filtered through a sterile 0.45 μm bottle filter and kept cold until it was needed. The sterility of the virus was verified through three rounds of serial passage on CEL cells, 5% CO_2_ incubation for seven days without causing cytopathic effect (CPE), and negative PCR amplification of the hexon gene. Inactivated FAdV and filtered Montanide ISA 71VG adjuvant were completely combined in a sterile bottle at a ratio of 1:1 (v/v) at room temperature, vortexed for 2 h, and kept at 4°C until needed.

### Ethical statement

The experiments in this study involving the use of animals, including the embryonated chicken egg utilization protocols, were carried out according to the guidelines and ethics of the Institutional Animal Care and Use Committee (IACUC) of Universiti Putra Malaysia, which was approved with ref number UPM/IACUC/AUP-R086/2018.

### Experimental design and sampling for immunogenicity, safety, and efficacy of inactivated FAdV serotype 8b isolate UPM08136CEL5B1 on commercial broiler chickens

Ninety-two one-day-old commercial broiler chicks were shared into two groups at random: group A (control) had 36 chicks, while group B (FAdV-inoculated) had 56 chicks (Table 1). The groups were split up as follows: B3 had 16 chickens, A2, B2, and B4 had 8 chicks apiece, and A1 and B1 had 28 and 24 chicks, respectively. Unlimited access to food and water was provided, and for 42 days, clinical symptoms were noted every day. While chicks in group A received no vaccination, all of the chicks in group B received a subcutaneous injection of 0.5 ml of an inactivated FAdV isolate of UPM11142CEL5B1 (108.3 TCID_50_/ml). Chicks in groups B3 and B4 received a booster dose of 0.5 ml of inactivated FAdV subcutaneously at 14 days post-infection. As a challenge, chickens in groups A2, B2, and B4 (each containing eight birds) received 0.5 ml of pathogenic FAdV of UPM11142CEL5EP2 (108 TCID_50_/ml) by intramuscular injections on day 28 pi. As shown in Table 1, four chickens from each group were sampled on each sampling day. The liver-body weight (BW) ratio was computed from recorded BW and liver weight. Gross lesions were recorded. From each chicken, liver, thymus, and spleen were sampled for histopathological alterations and immunophenotyping; serum was taken for FAdV antibody titer; and liver and cloacal swabs were taken for FAdV load and shedding detection and quantification, respectively.

**Table 1.** Experimental design for immunogenicity and efficacy of BEI inactivated FAdV serotype 8b isolate UPM11142CEL5B1 adjuvanted with Montanide 71VG on commercial broiler chickens

Groups	Time (day post inoculation (dpi))							
	0+	7	14*	21	28#	35	42	Total
A1	4	4	4	4	4	4	4	28
A2						4	4	8
B1	-	4	4	4	4	4	4	24
B2	-	-	-	-	-	4	4	8
B3	-	-	-	4	4	4	4	16
B4	-	-	-	-	-	4	4	8
Total								92

All chicks in B group were inoculated with the inactivated FAdV at day old of age (+). Booster was given to the booster group of chickens (B3 and B4) at day 14 dpi (*). The chickens in the challenge groups A2, B2, and B4 were challenged with pathogenic FAdV at day 28 dpi (#).

### Gross lesions and histological changes

Samples of the liver, spleen, and thymus from each chicken were observed for gross lesions before formalin fixing for 48 h and tissue processing. After processing onto glass slides, tissues were stained with hematoxylin and eosin (HE), examined under a light microscope (Leica DM LB2), and stained tissue images captured with Leica DFC295.

### FAdV antibody analysis by ELISA

The serum samples collected from each chicken were analyzed for FAdV antibody using an ELISA kit (BioChek, UK) at 405 nm on an ELISA reader (Dynatech MR7000, USA) following the recommended protocol.

### Immunophenotyping by flow cytometry

On every sample day, samples from every chicken in various groups were gently macerated, then filtered through a 70-μm cell strainer (FALCON-Corning, NC, USA) into a centrifuge tube and centrifuged for five minutes at 2,000 rpm. The cells in 1 ml of PBS suspension of the pellets were counted. After being aliquoted to a Falcon tube (FALCON-Corning, NC, USA), the cells corresponding to 1×10^6^/ml from each sample were stained with mouse anti-chicken CD3-FITC, mouse anti-chicken CD4-APC, and mouse anti-chicken CD8α-PE antibodies (SouthernBiotech, Birmingham, AL, USA). After that, the cells were suspended in 500 μl of PBS for CD3+, CD4+, and CD8+ phenotyping using 10,000 live cells on a BD FACS Calibur flow cytometer (BD Biotec, San Diego, CA, USA). The cells were then rinsed 3x with PBS (PH7.4, 0.01M, 4°C). The data produced were analyzed with Cell Quest software (BD Biotec.).

### Primers and probes

The UPM11142CELP3EP2 challenge virus’s partial sequence was used to design the FAdV 8b qPCR primers (qHex-F 5’-GTT AGA CAC CAC CGC ACA GA-3’ and qHex-R 5’-GTC ACG GAA CCC GAT GTA GT-3’) and probe (qHex Probe 5’-FAM/C CCT CCT TCT GAG TAC GGA GAG-3’ BHQ1), which were specific for the challenge virus.

### Extraction of genomic DNA

Following the manufacturer’s instructions, 200 μl of the supernatant from each liver and cloacal swab sample was used to extract total DNA using an innuPREP viral DNA kit (Analytikjena, Germany), and a spectrophotometer was used to ascertain the concentration and purity of the extracted DNA.

### Generation of the standard curve and qPCR amplification

**Figure 1.**
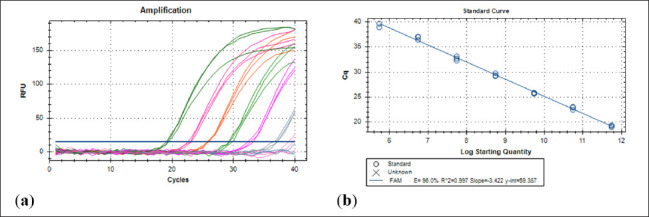
Amplification plot (a) and standard curve (b) of probe-based qPCR of viral genome copy number in the liver and cloacal swab of chickens infected with FAdV 8b challenge virus. Seven-fold dilution (100–0.0001 ng). The efficiency was 0.96 (96%), the regression squared value was 0.997, the slope was 3.422 and *y*-intercept was 59.387.

The standard curve was created using a FAdV positive control whose initial DNA content was 100 ng/μl and which was diluted seven times from 100 to 0.0001 ng/μl. The qPCR reaction was based on the template created from each liver sample and cloacal swab. This was done in a CFX96^TM^ Real-Time PCR Detection System (Bio-Rad, USA) in a 20 μl PCR reaction mix, which included 4 μl of template, 0.8 μl of primer pair, 0.2 μl of probe, 4.2 μl of nuclease-free water, and 10 μl of SensiFAST^TM^ Probe No-ROX Kit (Bioline, London, UK). For the non-template control, these dilutions and nuclease-free water were amplified three times each. The qPCR amplification settings were: initial denaturation at 95°C for 2 min; 40 cycles of denaturation; and extension at 95°C for 5 sec and 60°C for 20 sec. The amplification plot (Fig. 1a) and standard curve, as presented in Figure 1b, have an efficiency of 96%, a R square of 0.997, a slope value of 3,420, and a *y*-intercept of 26.008. Every replicate’s CT was acquired, and each sample’s mean was ascertained.

### Statistical analysis and data presentation

A 2-way repeated measures ANOVA on SPSS 25.0 for Windows (SPSS Inc., USA) was used to compare the differences within and between groups at a 5% probability level and means separated with Turkey HSD post hoc test. The results obtained were presented in tables and histograms.

## RESULTS

### Propagation in a bioreactor, inactivation, and challenge virus preparation

Six hundred milliliters of virus inoculum (10^8.3^ TCID_50_/ml) was realized after bioreactor propagation to produce UPM11142P5B1. The FAdV isolate was completely inactivated after 30 h of incubation with BEI. The challenge virus was serially passaged 2x in SPF embryonated chicken eggs to produce UPM11142P5EP2 (10^8^ TCID_50_/ml).

### Clinical signs and gross lesions

The chickens in the control-challenged group showed signs of depression and inappetence within 2 days after the challenge, which were not seen in other groups. At 42 days post-inoculation (dpi), two chickens had swollen, pale, and discolored livers, and two had enlarged spleens. Additionally, one and two chickens in the control-challenged group had enlarged thymus at 35 and 42 dpi, respectively, which were not observations in the organs of challenged and unchallenged birds in the booster and non-booster groups.

### The body, liver weight, and liver-to-BW ratio

Throughout the study, there was no statistically significant difference between the BW, liver weight, and liver: BW ratio of the hens in the non-booster and non-challenged booster groups and the uninoculated control group. But at day 21 pi and day 42 dpi, the BW of the challenged chickens was considerably higher (*p* < 0.05) than that of the booster and non-booster chickens. At 35 dpi, the liver-to-BW ratio was considerably larger (*p* < 0.05) in the unvaccinated, non-challenged hens (Table 2).

### Histopathological changes

**Figure 2.**
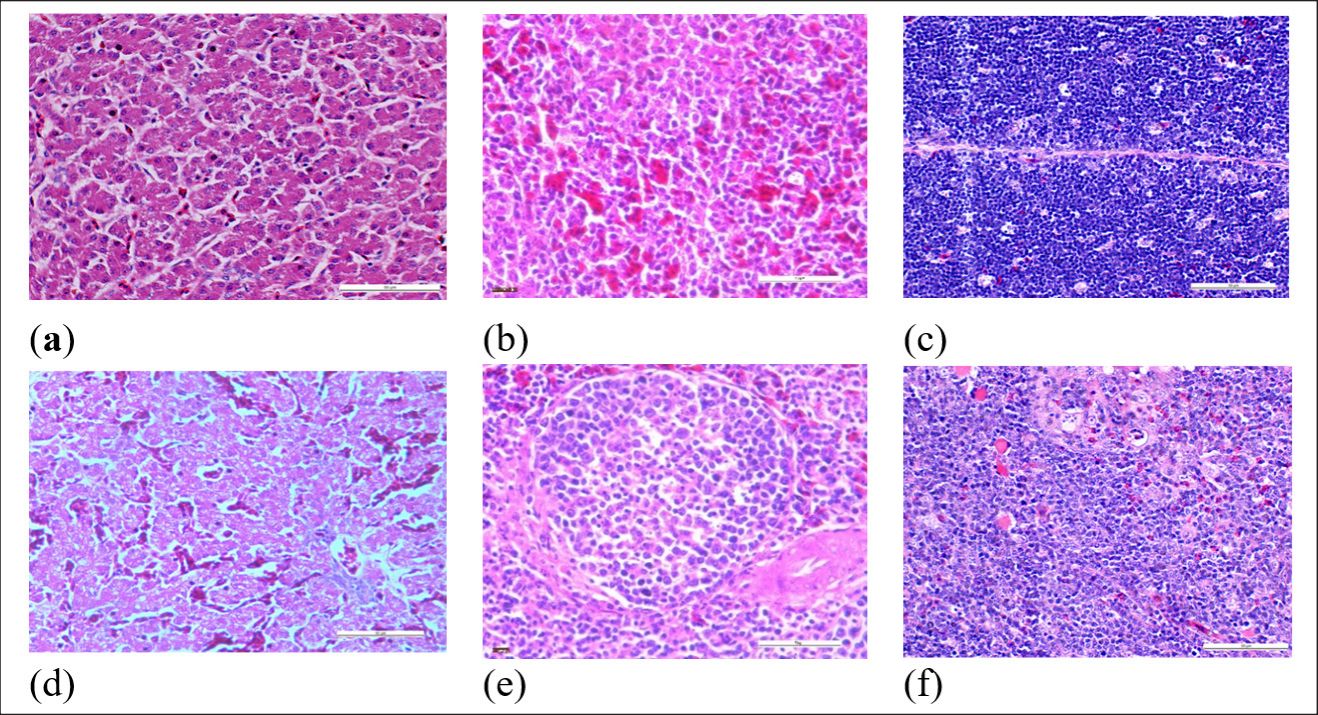
Microscopic images of the liver, spleen, and thymus of chickens inoculated with inactivated FAdV isolates UPM11142P5B1 and challenged. (a)–(c) shows the liver, spleen, and thymus of inoculated challenged chickens respectively with normal conformity, and (d)–(f) shows liver, spleen, and thymus of uninoculated control challenged chickens with necrosis, congestion, and vacuolation of the hepatocytes; cellular vacuolation and necrosis in spleen and lymphoid cells depletion in thymus respectively at 35 dpi. HE, 40x.

The challenged uninoculated control chickens showed necrosis, congestion, vacuolation, and hemorrhages in the liver; congestion, vacuoles with nuclear debris in the spleen and reduced thickness of the cortex; cell depletion in the medulla; and signs of lymphoid depletion in the thymus (Fig. 2d–f). The organs of the chickens in the other groups, however, showed normal appearances (Fig. 2b–c).

### FAdV antibody titer

At day zero, the antibody titer was 5353 ± 769, suggesting a high level of maternally produced antibodies. The non-booster B1 group had antibody titers of 4518 ± 2804, 1124 ± 732, 1476 ± 582, and 1058 ± 120 at 7, 14, 35, and 42 dpi, respectively (Table 3). These values were higher than the uninoculated control group, indicating the immunogenicity of the inactivated UPM11142CEL5B1 with Montanide 71VG adjuvant in commercial chickens. In comparison to the uninoculated control and non-booster groups, the antibody titer of the infected chickens in the B2 booster group was significantly higher (*p* < 0.05) at 28 dpi and higher (*p* > 0.05) at 35 and 42 dpi, respectively.

**Table 2.** Body weight, liver weight and liver-body weight ratio of commercial chickens inoculated with inactivated FAdV 8b.

Body weight (g) day PI									
	0	7	14	21	28	35	35 (CH)	42	42 (CH)
Body weight									
A1	^1^46.5 ± 2.72	^1,2^229.5 ± 11.87^a^	^2,3^457.75 ± 7.45^b^	^3^649.75 ± 26.68^a^	^4^1144 ± 44.47^a^	^5^1698.25 ± 71.63^a^	^4,5^1502.25 ± 97.43^a^	^6^2606.75 ± 153.3^a^	^6^2392.75 ± 153.17^a^
B1		^1^221 ± 11.7^a^	^1^404.5 ± 15.5^a^	^2^956 ± 27.0^b^	^2^1254.25 ± 72.4^a^	^3^1916 ± 153.5^a^	^3^1876.5 ± 19.1^a^	^4^2506.75 ± 50.3^a^	^5^2765.5 ± 111.5^b^
B2				^1^809 ± 24.2^b^	^1^1161.75 ± 25.9^a^	^2^1915.5 ± 49.9^a^	^2^1713.25 ± 108.7^a^	^3^2832.75 ± 78.2^a^	^3^2842.5 ± 130.2^b^
Liver weight									
A1	^1^1.08 ± 0.04	^2^11 ± 1.08^a^	^2,3^14 ± 0.47^a^	^2,3^17.75 ± 1.31^a^	^3^22 ± 0.70^a^	^4^35.5 ± 1.5^a^	^4^33 ± 1.95^a,b^	^5^48.75 ± 1.93^a^	^5^49.25 ± 5.18^a^
B1		^1^9.75 ± 0.62^a^	^1^11.5 ± 0.86^a^	^2^24.75 ± 1.79^a^	^2^24.5 ± 0.64^a^	^3^39 ± 4.3^a^	^3^38 ± 1.47^b^	^3^44.5 ± 2.36^a^	^3^48 ± 3.08^a^
B2				^1^23.25 ± 3.85^a^	^1^22.75 ± 0.85^a^	^1,2^40.25 ± 2.5^a^	^1^28.25 ± 2.75^a^	^2^53.75 ± 7.59^a^	^2^48.75 ± 3.14^a^
Liver-body weight ratio									
A1	^1^23 ± 0.07	^4^5 ± 0.27^a^	^3^3 ± 0.09^a^	^2,3^2.7 ± 0.10^a^	^1^1.9 ± 0.10^a^	^1,2^2.1 ± 0.16^a^	^1,2^2.2 ± 0.13^b^	^1^1.9 ± 0.07^a^	^1,2^2.1 ± 0.28^a^
B1		^4^4 ± 0.10^a^	^3^3 ± 0.16^a^	^2,3^2.6 ± 0.16^a^	^1,2^2 ± 0.07^a^	^1,2^2 ± 0.24^a^	^1,2^2 ± 0.08^b^	^1^1.7 ± 0.06^a^	^1^1.7 ± 0.16^a^
B2				^2^2.9 ± 0.38^a^	^1,2^2 ± 0.10^a^	^1,2^2.1 ± 0.11^a^	^1^1.6 ± 0.14^a^	^1^1.8 ± 0.21^a^	^1^1.7 ± 0.07^a^

A1=uninoculated control; B1=UPM11142P5B1 inoculated, non-booster; B2=UPM11142P5B1 inoculated, booster. 1,2,3 superscripts that are different are significantly different (*p*<0.05) across row while a, b, and c superscripts that are different are significantly different across column. Same superscripts are not significantly different (*p*>0.05).

### Flow cytometric immunophenotyping

The inoculated booster and non-booster chickens produced T lymphocytes in response to the inactivated FAdV 8b. Compared to the uninoculated control group, the challenged chickens had significantly higher CD3+ T cells in their liver at 14 and 28 dpi (*p* < 0.05), and at 35 and 42 dpi (*p *> 0.05). At 21 and 35 dpi and 14 and 28 dpi, respectively, it was greater (*p* > 0.05) in the thymus and spleen (Table 4). While the CD8+ T lymphocytes were significantly higher (*p* < 0.05) in the liver at 14 and 28 dpi, in the spleen at 21 dpi, and in the thymus at 21 and 28 dpi than in the uninoculated control chickens. There were higher CD4+ cells in the liver, spleen, and thymus at 7, 28, and 42 dpi; 14 and 21 dpi; and 7, 21, and 28 dpi, respectively (Table 4). Throughout the study, the T lymphocyte counts of the chickens in the booster and non-booster groups were statistically similar.

### FAdV challenges virus load in the liver and shedding in the cloaca

The copy number of the challenge virus in the cloaca of challenged uninoculated control chickens was considerably higher (*p *< 0.05) at 35 and 42 dpi (7 and 14 days post-challenge, respectively), compared to chickens in the booster and non-booster groups (Table 4). Compared to booster chickens at 42 dpi, the copy number in the cloaca of non-booster chickens was higher (*p* > 0.05) and similar at 35 dpi.

**Figure 3.**
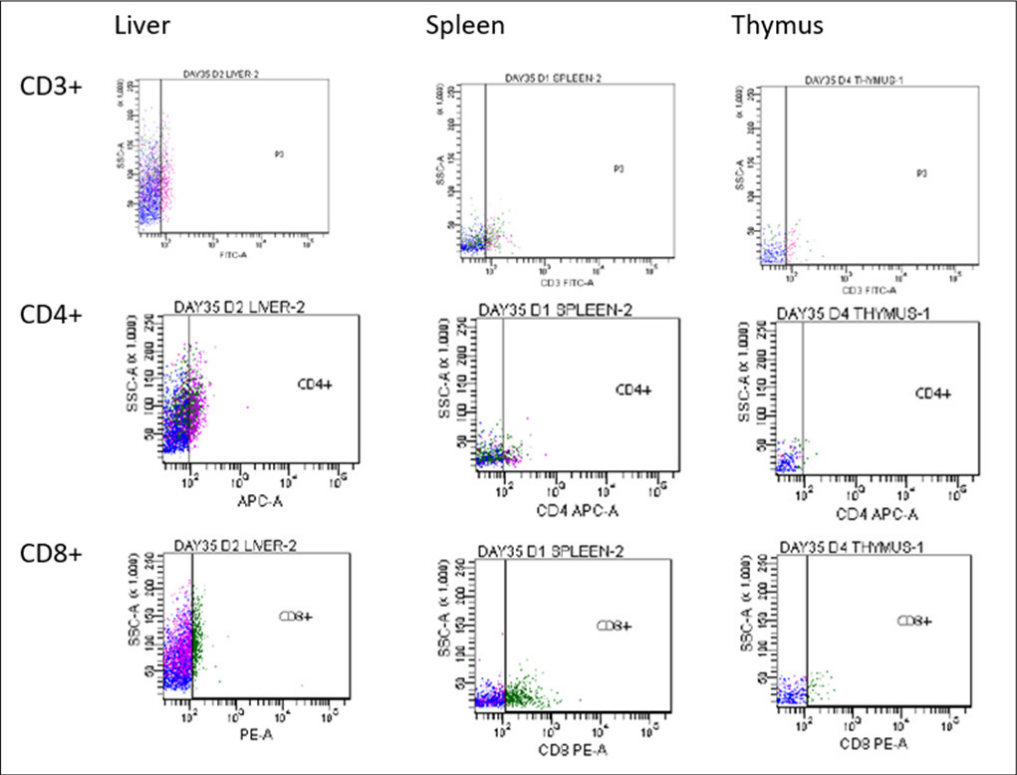
Dot-plots of the flow cytometric analysis of CD3+, CD4+, and CD8+ sub-population in the liver, spleen, and thymus of challenged chickens inoculated with inactivated FAdV 8b UPM11142P5B1 at 35 dpi.

According to the amplification plot (Fig. 3), the uninoculated control challenged group had a higher FAdV 8b challenge virus copy number in the liver at 35 dpi and a significantly higher (*p* < 0.05) copy number at 42 dpi than the chickens in booster and non-booster groups (Table 4). This indicates a decrease in the challenge virus’s proliferation in the liver of the inoculated chickens. When compared to booster chickens, the copy number in the liver of non-booster chickens was lower (*p* > 0.05) at 35 dpi and significantly lower (*p* < 0.05) at 42 dpi.

## Discussion

The Montanide 71VG adjuvanted inactivated FAdV 8b induced both humoral and CMI in the commercial broiler chickens, which was efficacious against pathogenic FAdV and reduced viral shedding. BEI inactivated FAdV serotype 4 with better results than formalin [15] and has proven to be a good inactivating agent against FAdV 8b.

Montanide 71VG adjuvant reported to have lesser side-effects and high immunopotentiation capacity, and which induces both humoral and cell-mediated immune (CMI) responses [16] could have contributed to the positive results in this trial and could be perfect for inactivated FAdV 8b since humoral immunity may not be the only requirement to control FAdV 8b infections in chickens [4].

**Table 3.** FAdV antibody titre of commercial chickens inoculated with inactivated FAdV 8b.

Antibody titre day PI									
	0	7	14	21	28	35	35 (CH)	42	42 (CH)
A1	^4^5353 ± 769	^1,2,3^350 ± 122^a^	^1,2,3,4^692 ± 300^a^	^1^84 ± 28^a^	^1,2^375 ± 181^a^	^1,2^198 ± 62^a^	^2,3,4^2115 ± 1588^a^	^1,2,3^612 ± 226^a^	^3,4^2983 ± 507^a^
B1		^2^4518 ± 2804^b^	^1,2^1124 ± 732^a^	^1^14 ± 9^a^	^1^245 ± 58^a^	^1,2^1476 ± 582^a^	^1,2^1651 ± 394^a^	^1,2^1058 ± 120^a^	^1,2^1382 ± 444^a^
B2				^1^115 ± 20^a^	^1,2^1905 ± 612^b^	^1,2^1576 ± 300^a^	^2^2473 ± 335^a^	^1,2^1671 ± 663^a^	^1,2^1193 ± 360^a^

A1=uninoculated control; B1=UPM11142P5B1 inoculated, non-booster; B2=UPM11142P5B1 inoculated, booster. 1,2,3 superscripts that are different are significantly different (*p*<0.05) across rows while a, b, and c superscripts that are different are significantly different across column. Same superscripts are not significantly different (*p*>0.05).

The high maternal antibodies (Mab) recorded at 0 dpi could be due to the prevalence of FAdV 8b in Malaysia [17, 18], which could have led to breeders transferring protection to their progenies after hatch. However, the Mab declined significantly at 7–21 dpi, which is in line with a previous report [19]. Although Mab is beneficial in chickens immediately after hatch, they suppress vaccine-induced antibody responses at a young age [19]. An Mab titer of 3433 recorded during vaccination caused a lag in the inducement of neutralizing antibodies in chickens [20]. This may have been caused by the ability of Mab to block B cell activity through the physical blocking of epitopes (epitope masking), leading to suppression of the development of neutralizing antibodies [21].

All of the groups showed increased antibody titers upon challenge, but the inoculated challenged groups demonstrated a link between antibody titer and viral load, as exemplified by the inoculated groups showing reduced viral loads in the liver at 35 dpi and significantly lower loads at 42 dpi. At 42 dpi, the antibody titer decreased in the groups with lower viral load and replication, and increased in the groups with greater load and replication, demonstrating the neutralizing effects of inactivated UPM11142P5B1 with Montanide 71VG and its protective efficacy. The inactivated FAdV serotype 8b incorporated with Montanide 71VG has shown its effectiveness in inhibiting the proliferation of the challenge virus in the liver of the inoculated, indicating its potential as a vaccine.

**Table 4.** Copy number of FAdV 8b challenge virus in the liver and cloaca of challenged chickens in different groups.

	35DPI	42DPI
Liver		
A1	7.71 ± 0.07^bc^	8.03 ± 0.11^c^
B1	7.57 ± 0.10^b^	6.95 ± 0.08^a^
B2	7.63 ± 0.03^b^	7.64 ± 0.05^b^
Cloaca		
A1	8.54 ± 0.01^b^	8.17 ± 0.04^b^
B1	8.07 ± 0.03^a^	7.72 ± 0.05^a^
B2	8.07 ± 0.08^a^	7.61 ± 0.01^a^

A1=uninoculated control; B1=UPM11142P5B1 inoculated, non-booster; B2=UPM11142P5B1 inoculated, booster. 1,2,3 superscripts that are different are significantly different (*p*<0.05) across row while a,b,c superscripts that are different are significantly different across column. Same superscripts are not significantly different (*p*>0.05).

There was an intermittent upregulation in the CD3+, CD4+, and CD8+ T cell sub-population at different time points in this trial, which is indicative of T lymphocyte inducement, a crucial need for recovering from viral infections [22]. Montanide 71VG may probably have contributed to the inducement having been reported to have such an attribute [11]. Complete protection against viral infections requires CMI, particularly CD8+ cells that eliminate virus-infected cells. Therefore, a high-quality vaccine should stimulate sufficient cellular immunity [22]. This may help to explain the report of a good protective immune response of FAdV serotype 8b vaccinations, which was previously unclear due to varying levels of neutralizing antibody titer [23]. The liver of the inoculated chicken groups had significantly more CD3+ T cells at 14 dpi, and the booster group had significantly more at 28 dpi than the uninoculated control group; the inoculated groups also had more CD4+ T cells throughout the trial than the uninoculated control group. Following the declining mAb, the CD8+ T cells in the infected chickens grew proportionately, indicating that the liver was prepared with cytotoxic T cells for virus clearance. As a result, the FAdV load in the liver of the inoculated group was significantly lower. The inoculation of chicken groups resulted in the induction of CD3+, CD4+, and CD8+ T cells in the spleen and thymus, with varying degrees of up-regulation observed at different times. This suggests that the chickens were ready to combat the FAdV challenge. This report is unique since there is a lack of information on the inducement of CMI by inactivated FAdV 8b, which makes it impossible to compare with prior findings. This could serve as a baseline report for future research, as it is the first report to the best of our knowledge on the evaluation of inactivated FAdV with Montanide 71VG inducing T cells in the liver, spleen, and thymus.

Similar to other findings on FAdV infections where FAdV 9 was shed for fourteen days [24], the FAdV challenge virus in this study, was shed for up to two weeks following the challenge. However, in SPF chickens infected at day old by oral route, FAdV 4 was shed for 28 days [25] and FAdV 1 for 12 weeks [26]. At 35 and 42 dpi, the uninoculated challenged group shed a considerably higher amount of the FAdV challenge virus (*p* < 0.05) than all the inoculated challenged groups. This suggests that the inactivated FAdV inoculations, whether given with or without booster, were effective in inducing blocking immunity. The capacity of a vaccine to diminish or stop viral shedding in the environment is a sign of vaccine efficacy, and this ability should be used to evaluate the effectiveness of viral vaccinations [27]. While repeated immunization decreased the amount of H3N2 influenza virus shedding, it did not outperform a single vaccine [25]. This is consistent with the study’s conclusion that, despite the booster group having less shedding at 42 dpi than the non-booster group, the results were not statistically significant.

The record of no mortality in this trial is similar to low or no mortality reported among 14-day-old broilers infected with FAdV-8b through the intramuscular route [28] and other experimental inactivated FAdV-8b vaccine inoculations given to chickens [6,7,29]. The mild clinical signs and histopathological changes observed in the uninoculated challenged chickens were not observed in the inoculated challenged chicken groups, similar to the report involving pathogenic FAdV 8a infection [30]. This suggests that the inactivated FAdV 8b strains with Montanide 71VG adjuvant are safe for chickens and effective at providing protection.

The liver weight ratio, liver weight, and BW of the inoculated control chickens (with or without booster) did not differ significantly from those of the uninoculated control chickens. However, the uninoculated control chickens that were challenged had a significantly lower BW than the chickens that were not challenged, indicating that the FAdV challenge had stunted their growth, which is consistent with the poor growth rate of chickens infected with FAdV [17]. Once more, the inactivated FAdV with Montanide 71VG was efficacious, as evidenced by the significantly greater BW, liver BW ratio, and lower liver weight among the challenged inoculation chickens.

## Conclusions 

In conclusion, a FAdV isolate UPM11142CELP5B1 cultivated in a bioreactor was successfully inactivated and inoculated into broiler chickens with and without a booster. When administered alone on day 0 or in combination with a booster at 14 dpi, the BEI-inactivated UPM1142CELP5B1 mixed with Montanide 71VG, water in oil adjuvant, induced both humoral and CMI, protecting commercial broiler chickens against pathogenic FAdV challenge and reducing viral load in the liver and viral shedding. This means that the inactivated strain administered singly or with a booster may be effective as a candidate vaccine for the prevention and control of FAdV 8b infections in chickens.

## List of Abbreviations

IBH, inclusion body hepatitis; BG, booster group; NBG, non-booster group; UCG, unchallenged group; CCG, challenged control group; UVC, ultraviolent-C; CMI, cell-mediated immunity; FAdV, Fowl adenovirus; BEI, binary ethylene imine; CEL, chicken embryo liver; CPE, cytopathic effects; IACUC, Institutional animal care and utilization committee; PCR, polymerase chain reaction; TCID, tissue culture infective dose; ELISA, enzyme-linked immunosorbent assay; qPCR, quantitative polymerase chain reaction; ANOVA, analysis of variance; BW, body weight.
